# Creation of a Novel Nomogram Based on the Direct Bilirubin-To-Indirect Bilirubin Ratio and Lactate Dehydrogenase Levels in Resectable Colorectal Cancer

**DOI:** 10.3389/fmolb.2021.751506

**Published:** 2021-10-20

**Authors:** Yifei Ma, Lulu Shi, Ping Lu, Shuang Yao, Hongli Xu, Junjie Hu, Xin Liang, Xinjun Liang, Shaozhong Wei

**Affiliations:** ^1^ Department of Gastrointestinal Oncology Surgery, Hubei Cancer Hospital, Wuhan, China; ^2^ Colorectal Cancer Clinical Research Center of Hubei Province, Wuhan, China; ^3^ Colorectal Cancer Clinical Research Center of Wuhan, Wuhan, China; ^4^ Department of Abdominal Oncology, Hubei Cancer Hospital, Wuhan, China

**Keywords:** colorectal cancer, prognosis, nomogram, bilirubin, lactate dehydrogenase

## Abstract

**Background:** Recently, many studies have suggested that bilirubin is associated with the prognosis of colorectal cancer (CRC). Conversely, there is substantial evidence that lactate dehydrogenase (LDH) levels are associated with the prognosis of cancer. Therefore, we sought to find a novel marker based on the above to predict prognosis in patients with resectable CRC.

**Methods:** A total of 702 patients from Hubei Cancer Hospital were included. The whole population was randomly divided into training (*n* = 491) and testing (*n* = 211) cohorts. Next, we established a new index based on direct bilirubin, indirect bilirubin and LDH levels. Chi-square tests, Kaplan-Meier survival analyses, and Cox regression analyses were used to evaluate prognosis. The prediction accuracies of models for overall survival (OS) and disease-free survival (DFS) were estimated through Harrell’s concordance index (C-index) and the Brier score.

**Results:** The median DFS duration was 32 months (range: 0–72.6 months), whereas the median OS duration was 35 months (range: 0 months–73.8 months). In addition, a new indicator, (DIR.LDH) (HR: 1.433; 95% CI, 1.069–1.920) could independently predict outcomes in CRC patients. Moreover, the module based on DIR. LDH was found to have exceptional performance for predicting OS and DFS. The C-index of the nomogram for OS was 0.802 (95% CI, 0.76–0.85) in the training cohort and 0.829 (95% CI, 0.77–0.89) in the testing cohort. The C-index of the nomogram for DFS was 0.774 (95% CI, 0.74–0.81) in the training cohort and 0.775 (95% CI, 0.71–0.84) in the testing cohort.

**Conclusion:** We successfully established a novel module to guide clinical decision-making for CRC.

## Introduction

Colorectal cancer (CRC) is the second most common cause of cancer-related death in the United States. In 2020, there were approximately 147,950 new cases and 53,200 deaths (Siegel et al., 2020). In China, it is the fifth most commonly diagnosed cancer and the fifth most common cause cancer-related death. The incidence of CRC has risen over the past decade, making it the third most common cancer in men (12.8%) and women (11.3%) (Chen et al., 2019). CRC is the fifth leading cause of cancer-related death in men (8.0%) (the top three being lung, liver, and stomach cancer), whereas among women, it is the third leading cause (9.8%) (the top two being lung and stomach cancer) (Feng et al., 2019). At present, surgical treatment is still the most important and decisive method for the treatment of CRC, but the effect of single surgical resection is often not satisfactory. Radiotherapy and chemotherapy, immunotherapy, and targeted therapy are becoming increasingly important colorectal cancer treatment options. However, there are still few predictors of efficacy that can truly guide clinical decision making. Therefore, there is an urgent need to identify effective prognostic markers for the stratified management of cancer patients.

Bilirubin is the main metabolite of iron porphyrin compounds in the body. It is toxic and can cause irreversible damage to the brain and nervous system, but it also has antioxidant functions and can inhibit the oxidation of linoleic acid and phospholipids. Studies from the past decade have indicated that mildly elevated serum bilirubin levels are closely associated with a reduced prevalence of chronic diseases, such as cardiovascular disease (Wagner et al., 2015). Recent data have suggested that bilirubin levels are associated with cancer prognosis. Therefore, bilirubin levels, as a biomarker of some diseases, have important clinical significance.

Lactate dehydrogenase (LDH) is a well-known diagnostic marker for myocardial infarction, liver dysfunction and various types of myopathies (Wróblewski and Gregory, 1961; Mg and Mj, 1964; Kopperschläger and Kirchberger, 1996a). However, some researchers found that LDH levels were also elevated in cancer patients. Elevated LDH levels are associated with the recurrence and metastasis of several tumours, such as pancreatic carcinoma, non-small-cell lung cancer, hepatocellular carcinoma and CRC (Tas et al., 2001a; Danner et al., 2010).

Therefore, we attempted to combine serum bilirubin and LDH levels to explore a new prognostic marker for CRC patients and establish a prognostic model with resectable colorectal cancer patients.

## Materials and Methods

### Study Population

In total, 702 patients with histopathologically confirmed CRC who had undergone resection of the primary lesion at Hubei Cancer Hospital, Hubei, China, between January 2013 and December 2016 were enrolled in our retrospective study. The following baseline indicators were analysed: age, sex, family history, history of smoking and drinking, stage of TNM and some pathological conditions, including tumour differentiation, location, nerve infiltration status, circumferential margin status, and vascular cancer embolus status. Importantly, LDH, direct bilirubin (DBIL), indirect bilirubin (IDBIL), carcinoembryonic antigen (CEA), and carbohydrate antigen 19-9 (CA199) levels were also retrieved the week before surgery. The following inclusion criteria were used: 1) age ≥18 years; 2) primary CRC patients; and 3) patients who had radical surgery. The exclusion criteria were as follows: 1) cooccurrence of other cancers and 2) patients lacking clinical data. This study was supported by the Ethics Committee and Institutional Review Board of Hubei Cancer Hospital. In addition, all patients provided informed consent.

### Blood Sample Analysis

Routine preoperative blood examinations were performed within 1 week before surgery. The DIR was defined as the level of direct bilirubin divided by the level of indirect bilirubin. Next, we divided patients into a high-level group or a low-level group according to the cut-off values of the DIR and LDH levels. Patients with low DIR and LDH levels were assigned a score of 0, those with a high DIR level or a high LDH level were assigned a score of 1, and those with high DIR and LDH levels were assigned a score of 2.

We randomly divided the total sample into a training cohort (70%) and a testing cohort (30%). The training group was used to determine the cut-off values and establish the prediction model, while the validation group was used to test the performance of the new index and the prediction model.

### Statistical Analyses

The chi-square test or Fisher’s exact test was used to compare differences between groups. By using cut-off values obtained via receiver operating characteristic (ROC) curve analyses, continuous variables were transformed into categorical variables. The Kaplan-Meier method was used to establish the survival curves, and the log-rank test was used to analyse the differences between groups. Multivariate Cox regression analyses were performed to identify independent prognostic factors. Harrell’s concordance index (the C-index) and the Brier score were used to estimate the efficacy of the models. Time-dependent ROC curves, calibration curves, and nomograms were plotted to visualize the performance of the models. Differences with a two-tailed p value < 0.05 were considered statistically significant. The time-dependent ROC curve, calibration curve, and nomogram were generated using the “survival ROC,” “time ROC,” “pec” and “regplot” packages of R 3.6.0 (The R Foundation for Statistical Computing, Vienna, Austria).

## Results

### Pooled Population

Our study included a cohort of 702 patients with a diagnosis of CRC (clinical and pathological characteristics are listed in Table 1). In the whole cohort, 22.5% of patients had rectal cancer, and 59.6% had right-sided colon cancer. Approximately 64.1% of patients had negative lymph nodes. Of note, 13.6 and 75.6% of patients had poorly differentiated and moderately differentiated tumours, respectively. In addition, 10.8% of patients had stage 1 disease, 33.7% had stage 2 disease, and 36.4% had stage 3 disease. Moreover, 69.8% of patients received neoadjuvant chemotherapy, and 5.9% of patients received postoperative adjuvant radiotherapy. According to the ROC curve, the optimal cut-off values for the DIR and LDH levels were 0.42 and 221 (IU/L), respectively.

**TABLE 1:** Demographic and tumor characteristics of colorectal cancer patients.

		DIR.LDH			
		0	1	2	P
Age(years)	<65	353 (50.2)	93 (13.2)	15 (2.1)	0.389
	≥65	182 (25.9)	51 (7.3)	8 (5.5)	
Sex	Male	345 (49.1)	84 (12.0)	12 (1.7)	0.389
	Female	190 (27.1)	60 (8.5)	11 (1.6)	
Family history of CRC	no	469 (66.8)	122 (17.4)	20 (2.8)	0.461
	yes	66 (9.4)	22 (3.1)	3 (0.5)	
BMI(kg/m2)	<25	404 (57.5)	116 (16.5)	17 (2.4)	0.429
	≥25	131 (18.7)	28 (4.0)	6 (0.9)	
Smoking	No	351 (50.0)	107 (15.2)	21 (3.0)	0.007
	Yes	184 (26.2)	37 (5.3)	2 (0.3)	
Drink	No	414 (58.9)	27 (3.8)	21 (3.0)	0.122
	Yes	121 (17.2)	117 (16.7)	2 (3.37)	
Lymph node ratio	<0.07	353 (50.3)	84 (12.0)	13 (1.9)	0.176
	≥0.07	182 (26.0)	60 (8.5)	10 (1.3)	
Location	Left colon	87 (12.4)	37 (5.2)	5 (0.7)	<0.001
	Right colon	339 (48.3)	32 (4.6)	8 (1.1)	
	Rectal	109 (15.5)	39 (5.6)	10 (6.6)	
postoperation radiotherapy	No	504 (71.8)	134 (19.1)	22 (3.1)	0.845
	Yes	31 (4.4)	10 (1.4)	1 (0.2)	
TNM stage	1	63 (9.0)	11 (1.6)	2 (0.3)	<0.001
	2	194 (27.6)	39 (5.6)	4 (0.6)	
	3	211 (30.1)	44 (6.3)	1 (0.1)	
	4	67 (9.5)	50 (7.1)	16 (2.2)	
Cea (ng/ml)	<3.5	308 (43.9)	54 (7.7)	5 (0.7)	<0.001
	≥3.5	227 (32.3)	90 (12.8)	18 (2.6)	
Ca199 (ng/ml)	<35.5	413 (58.8)	94(13.4)	10 (1.4)	<0.001
	≥35.5	122 (17.4)	50 (7.1)	13 (1.9)	
Circumferential margin	No	527 (75.1)	140 (20.0)	21 (3.0)	0.027
	Yes	8 (1.1)	4 (0.6)	2 (0.2)	
Vascular cancer embolus	No	383 (54.6)	103 (14.7)	14 (2.0)	0.537
	Yes	152 (21.6)	41 (5.8)	9 (1.3)	
Nerve infiltration	No	412 (58.7)	121 (17.2)	17 (2.4)	0.273
	Yes	123 (17.5)	23 (3.3)	6 (0.9)	
Differentiation	Poor	68 (9.7)	19 (2.7)	9 (1.3)	0.032
	Moderate	408 (58.1)	110 (15.7)	13 (1.9)	
	Well	59 (8.4)	15 (2.1)	1 (0.1)	
neoadjuvant	No	166 (23.4)	39 (5.6)	7 (0.9)	0.658
	Yes	369 (52.6)	105 (15.0)	16 (2.5)	

Abbreviation: CEA, carcinoembryonic antigen; CA199, carbohydrate antigen 19-9; DIR, direct bilirubin/indirect bilirubin; LDH, lactate dehydrogenase; DIR.LDH, the combination of DIR and LDH.

### Training Cohort

**FIGURE 1:**
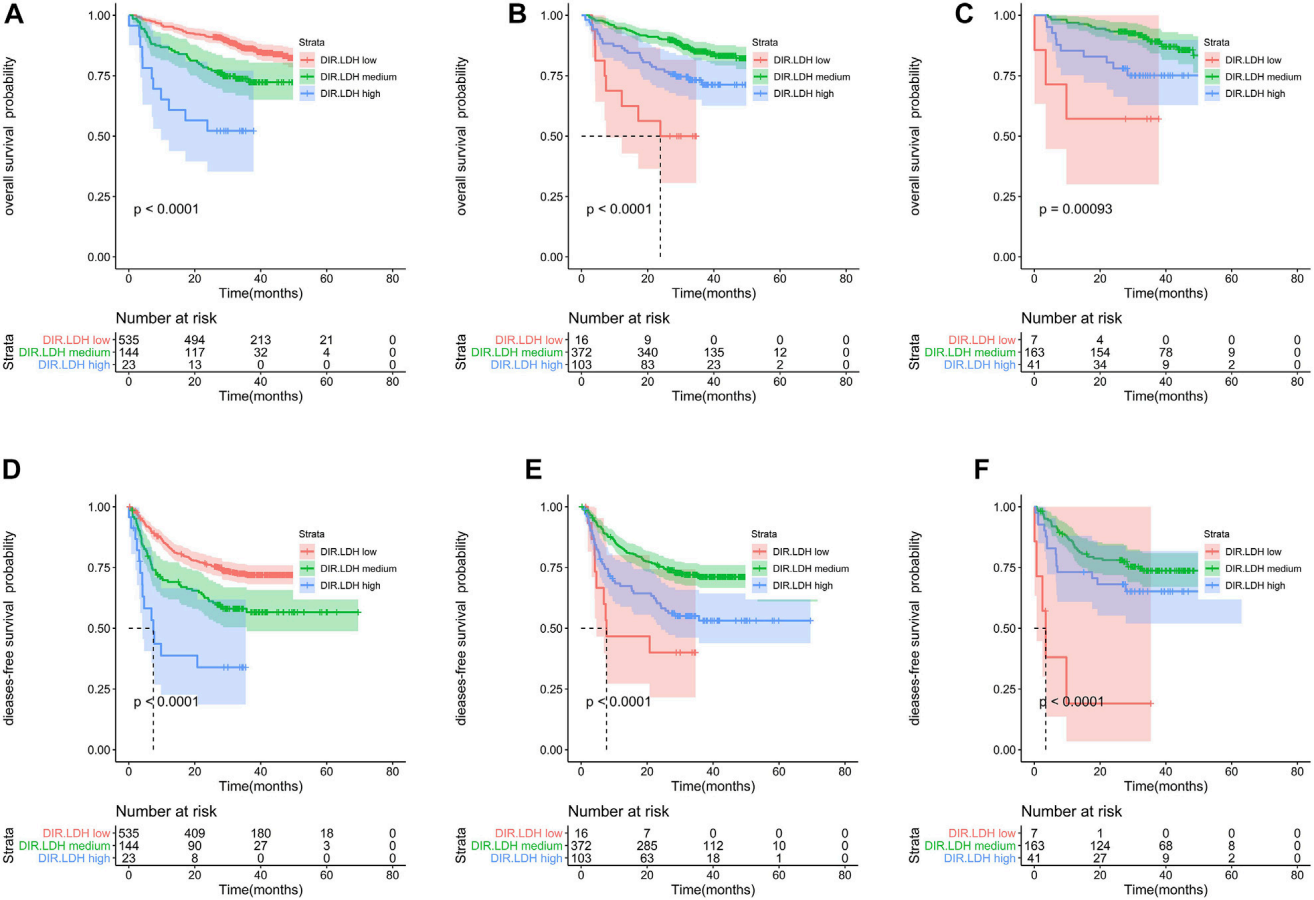
Kaplan-Meier survival analyses of DIR.LDH in the whole cohort **(A,D)**, training cohort **(B,E)** and testing cohort **(C,F)**. Abbreviations: DIR, direct bilirubin/indirect bilirubin; LDH, lactate dehydrogenase; DIR.LDH, the combination of DIR and LDH.

The training cohort included 491 patients. We found that OS and DFS differed in the total cohort, the training cohort or the validation cohort according to the DIR and LDH levels (DIR.LDH) (Figure 1). In addition, we found that the DIR. LDH was associated with the TNM stage (*p* < 0.001), the tumour location (*p* < 0.001), a history of smoking (*p* = 0.007), and CEA (*p* < 0.001) and CA199 levels (*p* < 0.001) (Table 1). In contrast, differentiation was not associated with DIR.LDH. In the univariate regression analysis, we found that the DIR.LDH, lymph node ratio, differentiation, TNM stage, circumferential margin, vascular cancer embolus, nerve infiltration, and CEA and CA199 levels might be independent prognostic factors for DFS and OS, and we included the above indicators in the multivariate regression analysis and found that DIR.LDH was indeed an independent prognostic factor for patients with CRC (Table 2). Next, we added the above significant indicators and some baseline indicators to our nomogram plot. Finally, we established two prognostic models for OS and DFS based on DIR.LDH.

**TABLE 2:** Univariate and multivariate analyses of the factors affecting overall survival and disease-free survival by Cox proportional hazard model.

			Disease-free survival				Overall survival			
Characteristic		N	Univariate analysis HR (95%CI)	P	Multivariate analysis^a^ HR (95%CI)	P	Univariate analysis HR (95%CI)	P	Multivariate analysis^b^ HR (95%CI)	P
Age(years)	<65	458	1	0.878	—	—	1	0.063	1	0.047
	≥65	244	0.978 (0.740–1.293)		—		1.398 (0.982–1.989)		1.435 (1.005–2.047)	
Sex	Male	444	1	0.339	—	—	1	0.429	—	—
	Female	258	0.873 (0.660–1.153)		—		0.861 (0.595–1.247)		—	
Family history of CRC	No	611	1	0.617	—	—	1	0.412	—	—
	Yes	91	0.908 (0.621–1.327)		—		1.262 (0.724–2.199)		—	
BMI(kg/m^2^)	<25	537	1	0.560	—	—	1	0.750	—	—
	≥25	165	0.910 (0.662–1.250)		—		0.935 (0.618–1.415)		—	
Smoking	No	479	1	0.952	—	—	1	0.821	—	—
	Yes	223	0.991 (0.747–1.315)		—		0.958 (0.660–1.390)		—	
Drink	No	552	1	0.206	—	—	1	0.774	—	—
	Yes	145	1.223(0.895–1.670)		—		0.938(0.609–1.447)		—	
Lymph node ratio	<0.07	450	1	<0.001	1	0.086	1	<0.001	1	<0.001
	≥0.07	252	2.819 (2.158–3.683)		1.283 (0.965–1.705)		4.774 (3.273–6.964)		2.503 (1.690–3.708)	
Location	Left colon	125	1	0.214	—	—	1	0.039	—	—
	Right colon	419			—				—	
	Rectal	158	0.904 (0.772–1.060)		—		0.808 (0.661–0.989)		—	
Differentiation	Poor	96	1	<0.001	1	0.011	1	<0.001	—	—
	Moderate	531							—	
	Well	75	0.513 (0.393–0.670)		0.693 (0.523–0.918)		0.568 (0.401–0.803)		—	
TNM stage	1	76	1	<0.001	1	<0.001	1	<0.001	1	<0.001
	2	237								
	3	256								
	4	133	2.296 (2.452–3.491)		2.180 (1.777–2.673)		3.166 (2.495–4.017)		2.307 (1.737–3.063)	
Cea (ng/ml)	<3.5	367	1	<0.001	1	0.013	1	<0.001	1	0.023
	≥3.5	335	2.586 (1.954–3.421)		1.466 (1.084–1.982)		2.869 (1.962–4.196)		1.588 (1.065–2.370)	
Ca199 (ng/ml)	<35.5	517	1	<0.001	1	0.002	1	<0.001	—	—
	≥35.5	185	2.639 (2.019–3.450)		1.579 (1.190–2.094)		2.678 (1.888–3.798)		—	
Circumferential margin	No	668	1	0.002	—	—	1	<0.001	—	—
	Yes	14	2.821 (1.446–5.503)		—		4.212 (2.056–8.628)		—	
Vascular cancer embolus	No	500	1	<0.001	—	—	1	0.274	—	—
	Yes	202	1.589 (1.206–2.094)		—		1.233 (0.847–1.794)		—	
Nerve infiltration	No	550	1	<0.001	—	—	1	0.029	—	-
	Yes	152	1.677 (1.253–2.245)		—		1.539 (1.046–2.266)		—	
postoperation radiotherapy	No	660	1	0.108	—	—	1	0.391	—	—
	Yes	42	1.501 (0.915–2.464)		—		1.346 (0.683–2.654)		—	
neoadjuvant	No	212	1	<0.001	—	—	1	0.224	—	—
	Yes	490	2.147 (1.524–3.025)		—		1.282 (0.859–1.914)		—	
DIR.LDH	0	535	1	<0.001	1	0.057	1	<0.001	1	0.016
	1	144								
	2	23	1.907 (1.526–2.383)		1.257 (0.993–1.590)		2.250 (1.710–2.960)		1.433 (1.069–1.920)	

a The multivariate Cox regression model included DIR.LDH, lymph node ratio, differentiation, TNM stage, circumferential margin, vascular cancer embolus, nerve infiltration, CEA, CA199 and neoadjuvant.

b The multivariate Cox regression model included DIR.LDH, lymph node ratio, location, differentiation, TNM stage, circumferential margin, nerve infiltration, CEA, CA199 and age.

Abbreviation: CEA: carcinoembryonic antigen; CA199: carbohydrate antigen 19-9; DIR: direct bilirubin/indirect bilirubin; LDH: lactate dehydrogenase; DIR.LDH: the combination of DIR and LDH.

**FIGURE 2:**
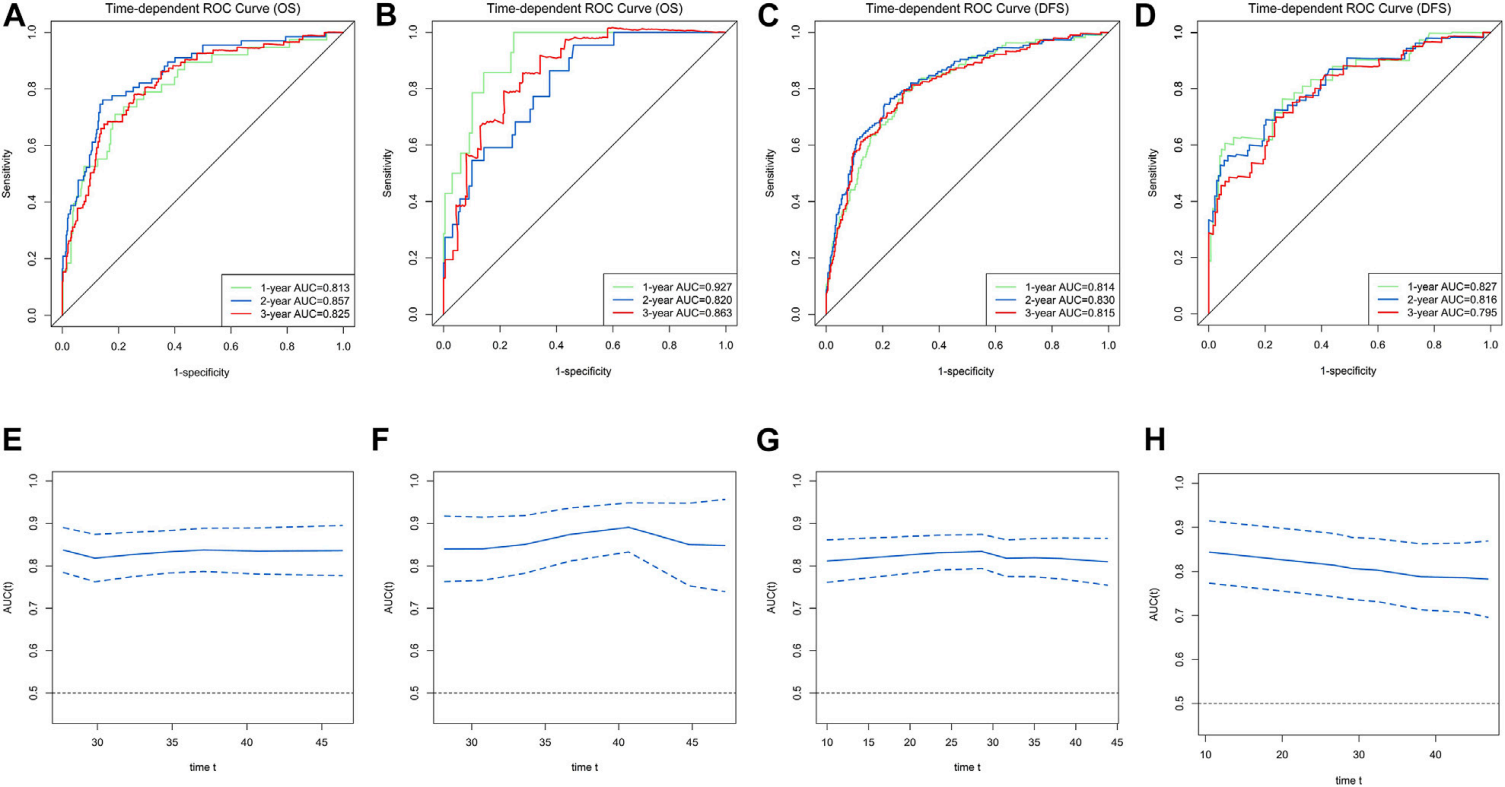
Time-dependent receiver operating characteristic (ROC) curves for the overall survival (OS) and disease-free survival (DFS)-associated nomograms for predicting 1-, 2- and 3-year survival rates and time-AUCs of the model. Time-dependent ROC curves from the nomograms for the prediction of OS and DFS rates in the training **(A,C)** and testing **(B,D)** sets. Time-AUCs from the nomograms for the prediction of OS and DFS rates in the training **(E,G)** and testing **(F,H)** sets.

**FIGURE 3:**
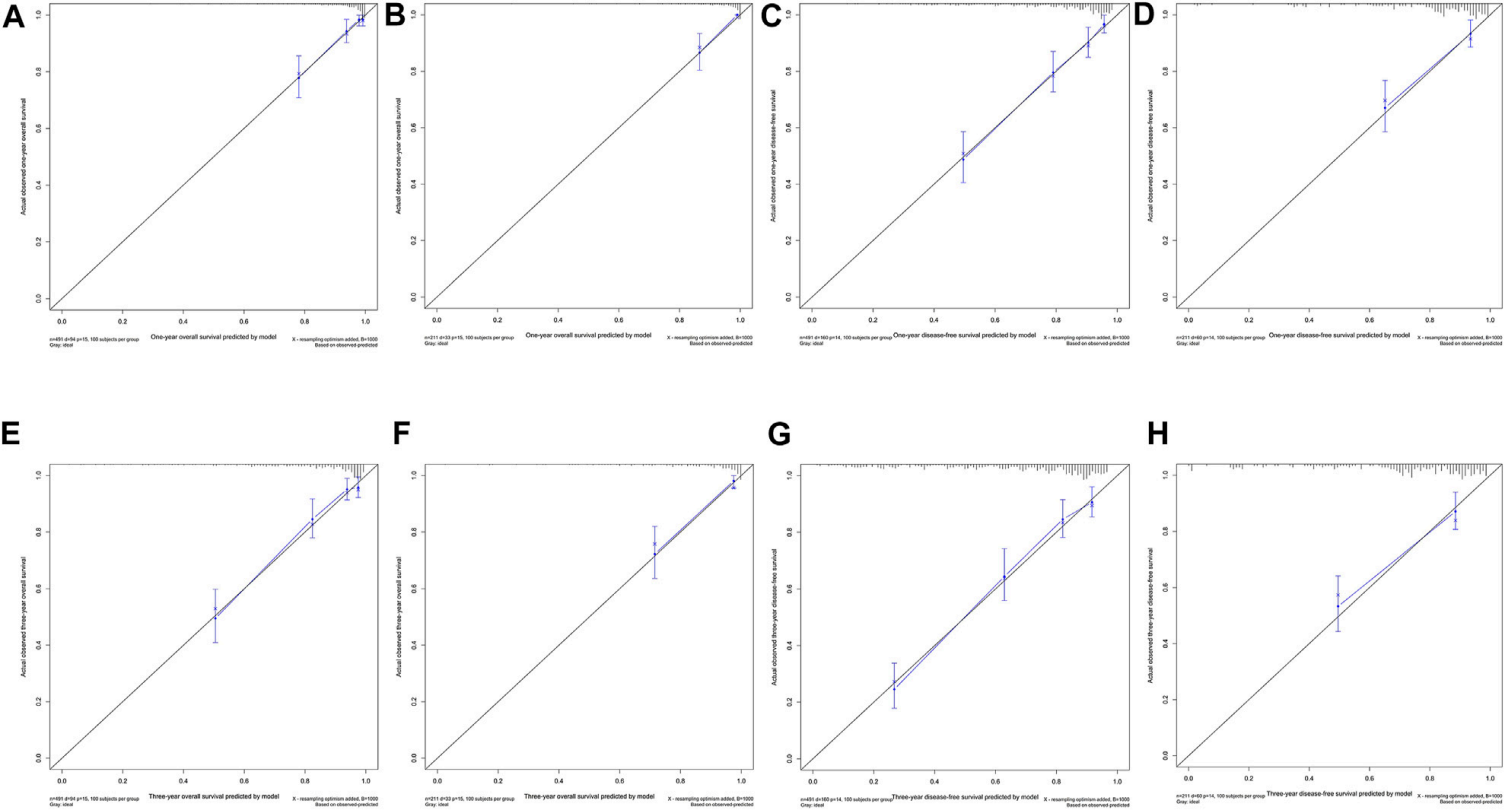
One-year and 3-year calibration curves of the model. One-year calibration curves from the nomograms for the prediction of OS and DFS rates in the training **(A,C)** and testing **(B,D)** sets. Three-year calibration curves from the nomograms for the prediction of OS and DFS rates in the training **(E,G)** and testing **(F,H)** sets.

Ultimately, age, sex, TNM stage, status of circumferential margin, status of vascular cancer embolus, status of nerve infiltration, differentiation, DIR.LDH, lymph node ratio, and CEA and CA199 levels were included in the nomogram. In the training cohort, the C-index of the prognostic model for OS based on age, sex, TNM stage, circumferential margin status, vascular cancer embolus status, nerve infiltration status, differentiation, DIR.LDH, lymph node ratio, location, CEA and CA199 levels was 0.802 (95% CI, 0.76–0.85). The C-index of the prognostic model for DFS based on age, sex, TNM stage, circumferential margin status, nerve infiltration status, differentiation, DIR. LDH, lymph node ratio, location, CEA and CA199 levels was 0.774 (95% CI, 0.74–0.81). We used one- and 3-year time-dependent ROC curves to determine the performance of the two models (Figures 2A,C). The area under the curve (AUC) values, which were 0.813 for 1-year OS and 0.825 for 3-year OS in the training set, were used to illustrate the predictive power of the two models. Furthermore, the AUC values for 1-year and 3-year DFS were 0.814 and 0.815 in the training set, respectively. In addition, the AUC values (95% CIs) of the two models were stable (Figures 2E,G), and the calibration curves showed good consistency between the predictions and observations in the 1-year and 3-year OS and DFS probabilities, with Brier scores of 0.034 and 0.069 for OS and 0.078 and 0.122 for DFS, respectively (Figures 3A,C,E,G).

**FIGURE 4:**
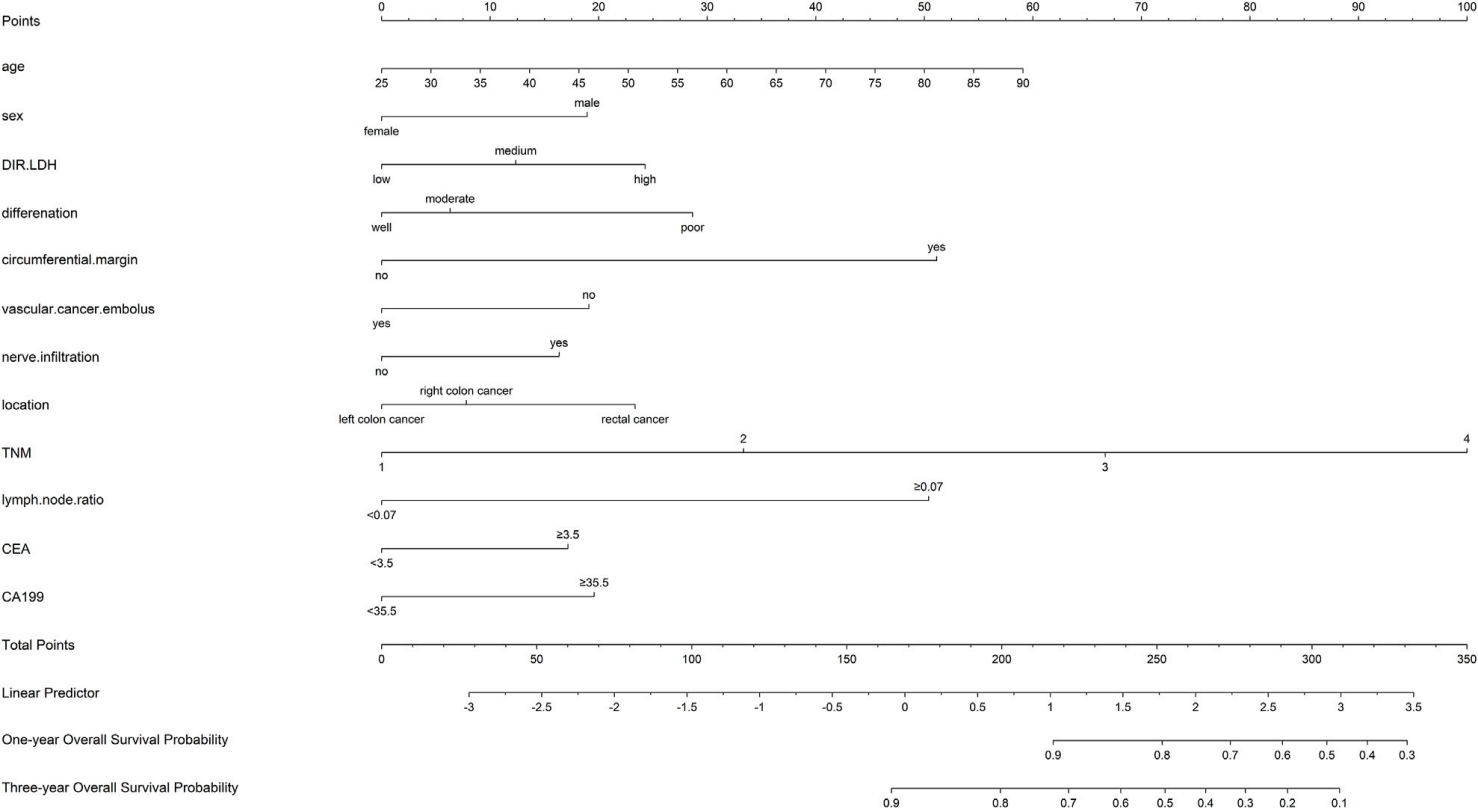
Nomogram of the prognostic model for the OS of patients with colorectal cancer.

**FIGURE 5:**
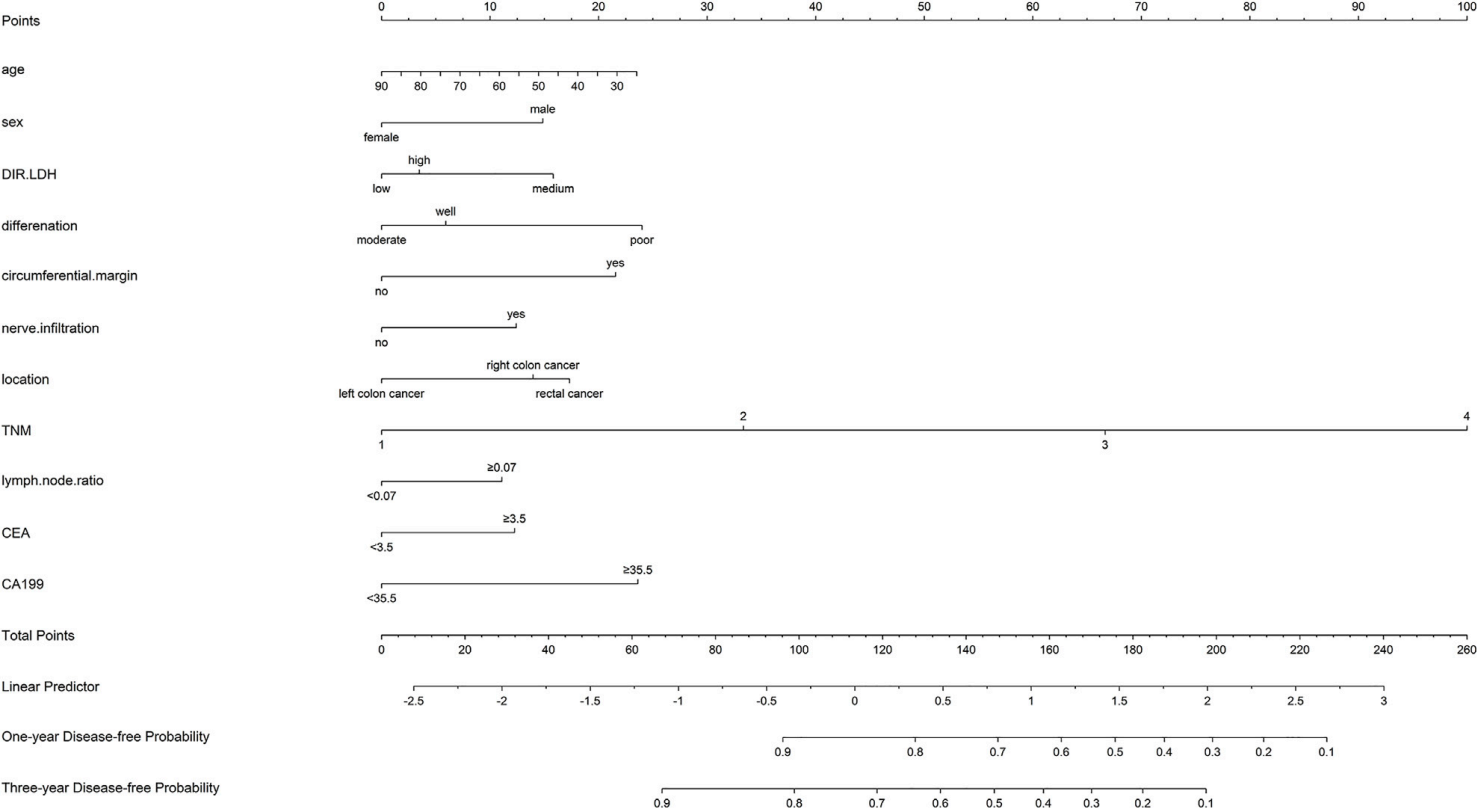
Nomogram of the prognostic model for the DFS of patients with colorectal cancer.

Nomograms for OS and DFS were plotted, as shown in Figures 4, 5, respectively.

### Testing Cohort

The testing cohort included 211 patients. The C-indices of the two models for DFS and OS were 0.775 (95% CI, 0.71–0.84) and 0.829 (95% CI, 0.77–0.89), respectively. One- and 3-year time-dependent ROC curves were used to verify the performance of the models (Figures 2B,D). Meanwhile, the AUC values of the 1-year and 3-year OS rates were 0.927 and 0.863 in the testing cohort, respectively. Furthermore, the AUC values of the 1-year and 3-year DFS rates were 0.827 and 0.795, respectively. The AUC values were stable over time (Figures 2F,H), and the calibration curves showed good consistency between the predictions and observations in the 1-year and 3-year OS and DFS probabilities, with Brier scores of 0.024 and 0.055 for OS and 0.070 and 0.113 for DFS, respectively (Figures 3D,F,H).

## Discussion

We found a new indicator, DIR. LDH, which was correlated not only with DFS but also with OS. More importantly, the prognostic model created with this metric in combination with other baseline values has high predictive performance for OS.

Bilirubin is involved in antioxidation and stress. Bilirubin inhibits complement induction by inhibiting the interaction between complement C1q and immunoglobulin, thereby inhibiting initial complement activation via classic pathways (Basiglio et al., 2010). However, few studies have reported that bilirubin is associated with the prognosis of CRC. It was reported that serum bilirubin was inversely related to total cancer mortality in a population-based cohort from Belgium (Temme et al., 2001). In addition, an association between baseline bilirubin levels and lung cancer has been reported (Horsfall et al., 2011; Wulaningsih et al., 2015). A real-world study showed that the cancer-related mortality risk of males linearly decreased as bilirubin increased, especially with reference to non-lung cancers (Temme et al., 2001). Another study showed that the relationship between unconjugated bilirubin (UCB) levels and CRC risk was related to sex, with high UCB levels being positively associated with CRC risk in men, and the opposite was true in women (Seyed Khoei et al., 2020). Therefore, our study combined the two and redefined new prognostic indicators, and the surprising findings have good prognostic value.

Some studies have shown that the glycolysis rate of malignant tumours is much higher than that of normal tissues in the tumour microenvironment, so the level of LDH will increase in malignant tumours. LDH can also participate in the formation of tumour blood vessels by mediating VEGF-A and VEGF receptor 1 overexpression (Tas et al., 2001b; Faloppi et al., 2016). Most importantly, LDH mediates immune escape from tumour cells by inhibiting immune function (Husain et al., 2013; Brand et al., 2016). Therefore, an increase in LDH levels through enzyme or gene regulation is considered to be beneficial to tumour growth (Certo et al., 2021). Moreover, for testicular cancer patients, LDH can be used to monitor patient outcomes and make decisions about therapeutic management (Hughes and Bishop, 1996; Shin and Kim, 2013). In the case of Wilms’ tumour, LDH is used as a marker in both diagnosis and monitoring the response to therapy (Schwartz, 1991; Kopperschläger and Kirchberger, 1996b; Pandian et al., 1997). Therefore, an elevated serum LDH level is an adverse prognostic factor for tumours.

In recent years, many studies have established prognostic models based on blood biomarkers. There is a very important reason why blood-based biomarkers are very reproducible, very quick to analyse and easy to use in clinical practice. We attempted to combine direct bilirubin, indirect bilirubin and LDH to explore a new predictor and establish a new prognostic model. Data analysis showed that our ideas are logical. A prognostic model based on age, sex, TNM stage, circumferential margin status, vascular cancer embolus status, nerve infiltration status, differentiation, DIR. LDH, lymph node ratio, location, CEA and CA199 levels was found to exhibit excellent predictive performance for OS [C-index: 0.802 (95% CI, 0.76–0.85) and Brier score: 0.034]. Another model based on age, sex, TNM stage, circumferential margin status, nerve infiltration status, differentiation, DIR.LDH, lymph node ratio, location, CEA and CA199 levels also exhibited excellent performance for DFS [C-index: 0.774 (95% CI, 0.74–0.81) and Brier score: 0.078]. More importantly, the model also showed good predictive performance in the testing sets. A previous model considering the mutation status and other parameters presented C-indices of 0.68 and 0.62 for progression-free survival (You et al., 2020).

There are some limitations to this study that should be mentioned. Most importantly, because this was a retrospective study, we did not include the genetic status of patients in our study. For RAS wild-type patients, there was a significant correlation between the efficacy of anti-EGFR mAb (cetuximab) and the tumour site, while no significant correlation between the efficacy of anti-VEGF mAb (bevacizumab) and the tumour site was observed. Cetuximab was superior to bevacizumab in objective response rate and overall survival in left colorectal cancer. In right-sided colon cancer, cetuximab is inferior to bevacizumab in terms of overall survival, although it may show an advantage with regard to the objective response rate (Tejpar et al., 2017). The 2020 NCCN recommended that patients with MSI-H/dMMR advanced colorectal cancer be treated with pembrolizumab and navurliumab (LE et al., 2015; Overman et al., 2016; Overman et al., 2018). Moreover, we did not consider the treatment modalities or the effects of targeted therapy and immunotherapy on prognostic markers. It is worth considering whether immunotherapy would have any effect on these blood-based indicators. In addition, due to the lack of relevant data, we regret that there is no external set to further verify our conclusions.

The advantages of this study are that we jointly considered changes in direct bilirubin, indirect bilirubin and LDH to establish two models that have excellent predictive performance, which has never been done before.

In conclusion, we innovatively combined the potential blood markers direct bilirubin, indirect bilirubin and LDH and further verified that our indicators were meaningful at different stages. Most importantly, we built a novel prognostic model based on direct bilirubin, indirect bilirubin and LDH to efficiently and practically predict the prognosis of CRC patients, and this model exhibited good predictive performance.

## Data Availability

The raw data supporting the conclusion of this article will be made available by the authors, without undue reservation.

## Abbreviations

AUC, area under the curve; CA199, carbohydrate antigen 19-9; CEA, carcinoembryonic antigen; C-index, Harrell’s concordance index; CRC, colorectal cancer; DBIL, direct bilirubin; DFS, disease-free survival; DIR, direct bilirubin to indirect bilirubin ratio; DIR.LDH, the combination of DIR and LDH; IDBIL, indirect bilirubin; LDH, lactate dehydrogenase; OS, overall survival; ROC, receiver operating characteristic curve; UCB, unconjugated bilirubin.
